# Transmissibility and pathogenicity of the emerging meningococcal serogroup W sequence type-11 complex South American strain: a mathematical modeling study

**DOI:** 10.1186/s12916-020-01552-7

**Published:** 2020-04-22

**Authors:** Matthieu Domenech de Cellès, Helen Campbell, Ray Borrow, Muhamed-Kheir Taha, Lulla Opatowski

**Affiliations:** 1 Université Paris-Saclay, UVSQ, Univ. Paris-Sud, Inserm, CESP, Anti-infective evasion and pharmacoepidemiology team, F-78180 Montigny-Le-Bretonneux, France; 2 Institut Pasteur, Epidemiology and Modelling of Evasion to Antibiotics, F-75015 Paris, France; 3 Max Planck Institute for Infection Biology, Charitéplatz 1, Campus Charité Mitte, 10117 Berlin, Germany; 4 Public Health England, NIS Immunisation and Countermeasures, London, England; 5 Public Health England Meningococcal Reference Unit, Manchester, England; 6 Institut Pasteur, National Reference Centre for Meningococci and Invasive Bacterial Infections Unit, Paris, France

**Keywords:** Meningococcal disease, Meningococcal carriage, Meningococcal serogroup W, Epidemiology, Transmissibility, Pathogenicity, Invasiveness, Mathematical modeling

## Abstract

**Background:**

The recent emergence of strains belonging to the meningococcal serogroup W (MenW) sequence type-11 clonal complex and descending from the South American sub-lineage (MenW:cc11/SA) has caused significant shifts in the epidemiology of meningococcal disease worldwide. Although MenW:cc11/SA is deemed highly transmissible and invasive, its epidemiological characteristics have not yet been quantified.

**Methods:**

We designed a mathematical model of MenW transmission, carriage, and infection to analyze the recent epidemiology of invasive disease caused by MenW:cc11/SA strains and by other MenW strains in England and in France. We confronted that model with age-stratified incidence data to estimate the transmissibility and the invasiveness of MenW:cc11/SA in England, using the data in France as a validation cohort.

**Results:**

During the epidemiological years 2010/2011–2014/2015 in England, the transmissibility of MenW:cc11/SA relative to that of other MenW strains was estimated at 1.20 (95% confidence interval, 1.15 to 1.26). The relative invasiveness of MenW:cc11/SA was also found to exceed unity and to increase with age, with estimates ranging from 4.0 (1.6 to 9.7) in children aged 0–4 years to 20 (6 to 34) in adults aged ≥ 25 years. In France, the model calibrated in England correctly reproduced the early increase of MenW:cc11/SA disease during 2012/2013–2016/2017. Most recent surveillance data, however, indicated a decline in MenW:cc11/SA disease. In both countries, our results suggested that the transmission of MenW:cc11/SA carriage possibly started several months before the first reported case of MenW:cc11/SA disease.

**Discussion:**

Our results confirm earlier suggestions about the transmission and the pathogenic potential of MenW:cc11/SA. The main limitation of our study was the lack of age-specific MenW carriage data to confront our model predictions with. Furthermore, the lesser model fit to the most recent data in France suggests that the predictive accuracy of our model might be limited to 5–6 years.

**Conclusions:**

Our study provides the first estimates of the transmissibility and of the invasiveness of MenW:cc11/SA. Such estimates may be useful to anticipate changes in the epidemiology of MenW and to adapt vaccination strategies. Our results also point to silent, prolonged transmission of MenW:cc11/SA carriage, with potentially important implications for epidemic preparedness.

## Background

The meningococcus (*Neisseria meningitidis*) is a bacterium that frequently colonizes the human nasopharynx, particularly that of adolescents and young adults [1]. Although colonization is typically asymptomatic and harmless to the host, the meningococcus can move from the nasopharynx to cause severe invasive diseases, such as bacteremia and meningitis [2]. The burden of invasive meningococcal disease (IMD) varies substantially worldwide, with the highest incidence rates observed in the so-called African meningitis belt, a region stretching from Senegal to Ethiopia that recurrently experiences large epidemics [3]. In contrast, the burden of IMD is generally low in high-income countries (e.g., annual incidence rate of 0.3–3/100,000 during 2004–2014 in Europe [4] and of 0.26/100,000 during 2006–2015 in the USA [5]), but the disease remains a public health concern because of its high lethality (typically 5–10%) and of its frequent long-term sequelae in survivors [6–8].

Although the *N. meningitidis* species is antigenically and genetically diverse, only six serogroups (A, B, C, W, X, and Y) cause the vast majority of IMD [9]. The genetic characterization of disease isolates using molecular typing methods has also demonstrated the predominance of a small number of clonal complexes (cc)—called hyper-invasive lineages—which can be associated with several serogroups [9, 10]. The epidemiology of IMDs caused by different serogroups and by different ccs displays substantial spatial heterogeneity worldwide [9]. Locally, temporal variations can also be pronounced and result in changes in the population structure of meningococci associated with disease [11]. Hence, close epidemiological surveillance has been essential to implement vaccination strategies, such as the introduction of the conjugate vaccine against the meningococcal serogroup C in 1999 in the UK [12].

Recent changes in the epidemiology of meningococcal serogroup W (MenW) provide another case in point [13]. Historically an infrequent cause of IMD, a large outbreak among Hajj pilgrims in 2000 led to an increase of MenW disease (in individuals returning from the Hajj or linked to those who had attended) in the early 2000s in different parts of the world, including Europe [14] and the USA [15]. Genomic analyses demonstrated the emergence of a new strain belonging to cc11—the MenW:cc11 Anglo-French Hajj strain [16]—that subsequently became a major cause of disease in the African belt, and elsewhere [17–19]. From the mid-2000s, an increasing incidence of MenW disease was also reported in a number of South American countries, starting with Brazil [20], followed by Argentina [21] and Chile [22]. A genetic comparison of the South American isolates with other MenW disease isolates revealed the emergence of a new strain also belonging to cc11, but distinct from the Hajj strain [16]. That strain subsequently spread worldwide and has caused increasing endemic disease in Europe [23, 24], in the USA [25], and in Australia [26]. In England, the South American strain and its descendants, collectively called the MenW:cc11 South American strain (MenW:cc11/SA) strain, caused a precipitous rise of MenW disease from 2009 [24, 27]. In response, an emergency program of vaccination with the MenACWY quadrivalent conjugate vaccine was implemented in adolescents from August 2015 [28]. Other countries that experienced comparable trends in MenW disease, such as the Netherlands, have also rolled out reactive MenACWY vaccination programs [13].

Because of its ability to spread and to cause severe disease, the MenW:cc11/SA strain is deemed highly transmissible and hyper-invasive [16, 29]. Quantitative estimates, however, are currently lacking, but are needed to anticipate changes in the epidemiology of MenW and to adapt vaccination strategies. Here, we analyzed the epidemiology of MenW in England and in France, both countries that recently witnessed an upsurge of cases caused by MenW:cc11/SA. Using mathematical transmission models, fitted to age-specific incidence data, we aimed to estimate the transmissibility (that is, the ability to transmit carriage from host to host) and the invasiveness (i.e., the risk of disease given carriage) of MenW:cc11/SA.

## Methods

### Data

Data in England were available from the Public Health England (PHE) Immunisation and Countermeasures Division which conducts nationwide surveillance of invasive meningococcal disease based on cases confirmed by the PHE Meningococcal Reference Unit [27, 30]. The data consisted of the case counts of laboratory-confirmed IMD during the epidemiological years (i.e., July to June) 2010/2011–2014/2015, stratified into different ccs (MenW:cc11/SA or other MenW strains not belonging to cc11 [non-MenW:cc11]) and into different age groups (< 1-, 1–4-, 5–14-, 15–19-, 20–24-, 25–49-, 50–64-, and ≥ 65-year-olds [yo]). Because the MenW:cc11 Hajj strain caused no reported case of disease during that period, MenW:cc11/SA accounted for all cases of MenW:cc11.

Data in France were provided by the National Reference Centre for Meningococci [31]. These data consisted of the age-stratified counts of non-MenW:cc11 IMD cases during 2010/2011–2016/2017 and of MenW:cc11/SA cases during 2012/2013–2018/2019 [23].

### Statistical analysis of trends and of age distributions

We used Poisson regression models with the log-transformed population sizes as offsets and the epidemiological year as a (quantitative) covariate to estimate the annual relative increase in the incidence of MenW:cc11/SA and of non-MenW:cc11 cases. To quantify the age distribution of cases, we used a binomial regression model that incorporated intercepts for MenW:cc11/SA and for non-MenW:cc11, age (as a categorical variable, 0–4, 5–24, and ≥ 25 yo) and an interaction term between age and MenW:cc11/SA. For all models, we calculated 95% confidence interval (CI) of every parameter using heteroskedasticity and autocorrelation consistent estimators of the covariance matrix [32, 33]. Finally, we used the delta method to estimate 95% CI of parameters derived from the model parameters [34].

### Mathematical model of MenW transmission, carriage, and infection

To analyze the recent epidemiology of disease caused by MenW, we formulated a mathematical model of transmission, carriage, and infection [35, 36], which represented the joint epidemiological dynamics of MenW:cc11/SA and of non-MenW:cc11. Because the epidemiology of meningococcus varies with age, the model was age-structured and incorporated age-specific contact rates, available from two empirical studies of self-reported contacts in Great Britain (Ref. [37, 38] and Additional file 1: Fig. S1). The model also incorporated age-specific risks of transmission and of invasion (i.e., progression from carriage to disease) to account for the known difference between carriage prevalence and disease incidence according to age (Ref. [1] and Fig. 2). To model the emergence of MenW:cc11/SA, we assumed that, from a given date *t*_0_, carriers of MenW:cc11/SA were imported from outside England or France and initiated the endemic spread of carriage. Because of the significant effect of stochasticity for emerging pathogens [39], we implemented the model as a continuous-time Markov process, approximated by a multinomial modification of Gillespie’s algorithm, with a fixed time step of 10^−3^ years [40]. We used the R pomp package (version 1.16) to implement the model and to estimate the model parameters [41, 42]. Complete details on the model formulation, including model equations, are provided in Additional file 1.

### Model parametrization in England

In England, we parametrized the model in two steps. First, because the epidemiology of non-MenW:cc11 was stable during the study period [24, 27], we calibrated the age-specific transmission risk and the age-specific invasion risk (i.e., the risk of invasive disease given carriage) so that the simulated dynamics of MenW were stationary before the emergence of MenW:cc11/SA (see Additional file 1). In the absence of longitudinal carriage data in every age group, we assumed that the carriage prevalence of non-MenW:cc11 was 2% in 20–24 yo [43] and varied over age as estimated in the meta-analysis of Christensen et al. [1] (Fig. 2a, b and Additional file 1). The values of other fixed parameters are listed in Table 1.

**Table 1:** Fixed model parameters in England and in France

Parameter	Symbol*	Value in England	Value in France	Source/comment
Birth rate	*B*(*t*)	Additional file 1: Fig. S2	Additional file 1: Fig. S2	Fixed so that simulated demography matched observed demography (see Additional file 1)
Demographic rates	*μ*_*a*_(*t*)	Additional file 1: Fig. S2	Additional file 1: Fig. S2	
Carriage prevalence of non-MenW:cc11	*c*_*a*_^(non-cc11)^	Fig. 2b	Fig. 2b	Fixed assuming a MenW carriage prevalence of 0.02 in 20–24 yo (Ref. [43], Table 1) Sensitivity analyses: alternative values of 0.01 and of 0.03 (Additional file 1: Table S1)
Transmissibility of non-MenW:cc11	*β*_*a*_	Fig. 2c	Fig. 2c	Calibrated to have stationary epidemiology of MenW before emergence of MenW:cc11/SA
Invasiveness of non-MenW:cc11	*θ*_*a*_	Fig. 2d	Fig. 2d	
Clearance rate of MenW carriage	*γ*	365/113 per year	365/113 per year	Average carriage duration of 113 days (Ref. [43], Appendix Table 3) Sensitivity analysis: value of 2 per year (Additional file 1: Table S1)
Age-specific contact rates	*m*_*a,a’*_	Additional file 1: Fig. S1	Additional file 1: Fig. S1	[37, 38]
Number of imported MenW:cc11/SA carriers	*ι*	10	10	Assumption, low value chosen to keep contribution of imported carriers to epidemiological dynamics minimal Sensitivity analyses: values of 1, 100 (Additional file 1: Table S1)
Time of MenW:cc11/SA carriage emergence	*t*_*0*_	2009.5	Estimated	First case of MenW:cc11/SA disease reported during season 2009/2010 in England Sensitivity analysis: value of 2008.5 in England (Additional file 1: Table S1)

*Symbols used in model equations, presented in Additional file 1

**Fig. 1:**
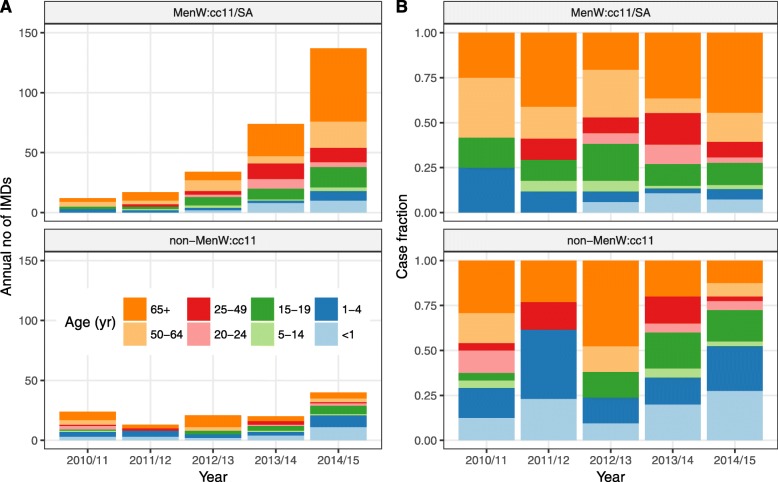
Incidence of MenW IMDs in England, 2010/2011–2014/2015. Annual number (**a**) and age distribution (**b**) of cases caused by MenW:cc11/SA and by other MenW not belonging to cc11 (non-MenW:cc11)

Second, we applied the iterated filtering algorithm [44] to estimate the relative (to non-MenW:c11) transmissibility and the relative invasiveness of MenW:cc11/SA, based on the observed case counts of MenW:cc11/SA IMD during 2010/2011–2014/2015 (Fig. 1). Specifically, we estimated four parameters:

1. The transmissibility of MenW:cc11/SA, relative to that of non-MenW:cc11, denoted by *r*^(*β*)^
2. The invasiveness of MenW:cc11/SA in children aged 0–4 years relative to that of non-MenW:cc11 in children aged 0–4 years, denoted by ${r}_I^{\left(\theta \right)}$
3. The invasiveness of MenW:cc11/SA in individuals aged 5–24 years relative to that of MenW:cc11/SA in 0–4 yo, denoted by ${r}_{II}^{\left(\theta \right)}$
4. The invasiveness of MenW:cc11/SA in ≥25 yo relative to that of MenW:cc11/SA in 5–24 yo, denoted by ${r}_{III}^{\left(\theta \right)}$

With this parametrization, the invasiveness of MenW:cc11/SA (relative to that of non-MenW:cc11 in the same age group) was equal to ${r}_I^{\left(\theta \right)}$ in 0–4 yo, to ${r}_I^{\left(\theta \right)}{r}_{II}^{\left(\theta \right)}$ in 5–24 yo, and to ${r}_I^{\left(\theta \right)}{r}_{II}^{\left(\theta \right)}{r}_{II I}^{\left(\theta \right)}$ in ≥ 25 yo. The estimation was completed in several steps, starting with trajectory matching to generate good starting parameter sets for the iterated filtering algorithm, followed by 100 independent runs to locate the maximum likelihood estimate. Finally, the profile log-likelihood was calculated to verify the convergence to the maximum likelihood estimates and to compute approximate 95% CI for each parameter [45]. The delta method was used to estimate 95% CI of parameters derived from the base model parameters, namely ${r}_I^{\left(\theta \right)}{r}_{II}^{\left(\theta \right)}$ and ${r}_I^{\left(\theta \right)}{r}_{II}^{\left(\theta \right)}{r}_{II I}^{\left(\theta \right)}$.

### Model parametrization in France

In France, we similarly calibrated the age-specific transmission and invasion risks, based on the observed annual counts of non-MenW:cc11 IMDs during 2010/2011–2016/2017. To verify the reliability of our estimates in England, however, we did not re-estimate the transmissibility and the invasiveness of MenW:cc11/SA. Rather, we estimated only the start time of MenW:cc11/SA carriage (i.e., the time at which the transmission of MenW:cc11/SA carriage started) by calculating the profile log-likelihood based on the MenW:cc11/SA incidence data during 2012/2013–2018/2019. To take into account the uncertainty of the parameter estimates in England, we conducted that estimation three times by considering three parameter sets along the likelihood profile of the transmissibility of MenW:cc11/SA.

## Results

### Emergence of MenW:cc11/SA in England

The overall incidence of MenW IMD increased markedly during the study period, from 0.07 cases per 100,000 in 2010/2011 to 0.32 cases per 100,000 in 2014/2015 (annual relative increase of 61 [35, 87]%, Fig. 1a). The incidence of MenW:cc11/SA increased by 92 [79, 105]% every year and almost entirely accounted for that trend. In contrast, the incidence of non-MenW:cc11 IMD remained relatively stable during the same period (relative variation of 17 [− 15, 50]% every year). Moreover, the age distribution of MenW:cc11/SA differed from that of non-MenW:cc11 (Fig. 1b), with a lower proportion of cases in children aged 0–4 years (14% vs. 41%, difference of − 27 [− 41, − 13]%) and a higher proportion of cases in adults aged ≥ 25 years (65% vs. 39%, difference of 26 [8, 45]%).

### Calibrated transmissibility and invasiveness of non-MenW:cc11 in England and in France

**Fig. 2:**
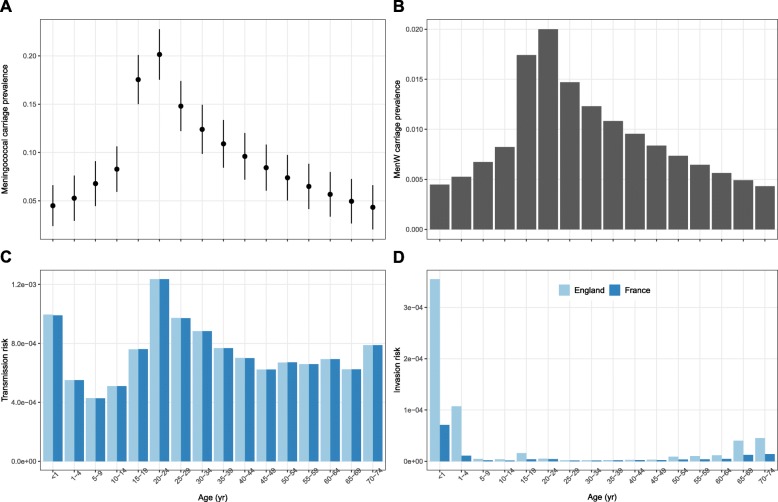
Fixed parameter values of MenW transmissibility and invasiveness before the emergence of MenW:cc11/SA in England and in France. **a** Age-specific overall prevalence of meningococcal carriage. The estimates and 95% CIs were estimated using weighted linear regression, based on the values reported in Ref. [1]. **b** Age-specific prevalence of non-MenW:cc11 carriage. **c**, **d** Age-specific transmissibility and invasiveness of non-MenW:cc11. We scaled the estimates of overall meningococcal carriage prevalence displayed in A to derive estimates of non-MenW:cc11 (that is, of MenW before the emergence of MenW:cc11/SA), assuming a carriage prevalence of MenW of 2% in 20–24 yo. We then calibrated the transmission risk (**c**, see Additional file 1) so that the carriage prevalence of non-MenW:cc11 was as displayed in **b**. Finally, we calibrated the invasion risk of non-MenW:cc11 (**d**) so that the simulated incidence of MenW was approximately equal to that reported in England or in France before the emergence of MenW:cc11/SA

The age-specific values of non-MenW:cc11 transmissibility and invasiveness—calibrated to reproduce the stationary epidemiology of MenW before the emergence of MenW:cc11/SA—are displayed in Fig. 2c and d. The variations of transmissibility and of invasiveness over age were W-shaped, with 3 local peaks in infants, in young adults, and in the elderly. Relative to young adults, however, the peaks in infants and in the elderly were much more pronounced for invasiveness than for transmissibility. These variations reflected the large incidence of reported disease, in contrast to the low carriage prevalence, in infants and in the elderly. Because the incidence of reported disease was higher in England than in France (average annual incidence rate ratio ranging from 1.2 to 9.8, depending on age), the calibrated values of invasiveness were correspondingly higher in England.

### Estimated transmissibility and invasiveness of MenW:cc11/SA in England

**Fig. 3:**
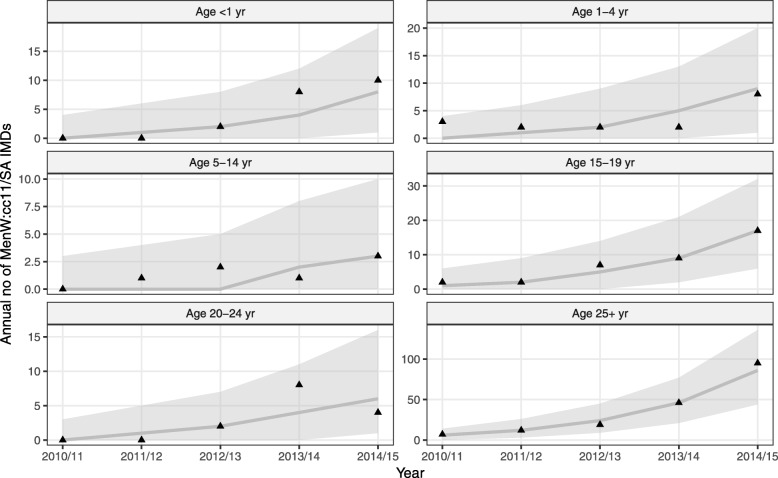
Model fit to MenW:cc11/SA age-stratified annual incidence data during 2010/2011–2014/2015 in England. The solid lines (envelope) represent the median (95% interval) simulated values from 2000 model simulations; the solid triangles represent the observed data

The transmissibility of MenW:cc11/SA relative to that of non-MenW:cc11 was estimated at 1.20 (95% CI [1.15, 1.26], Table 2). Equivalently, MenW:cc11/SA was estimated to be 20 [15, 26]% more transmissible than non-MenW:cc11. The invasiveness of MenW:cc11/SA relative to that of non-MenW:cc11 was also found to exceed unity and to increase with age, with estimates of 4.0 [1.6, 9.7] in children aged 0–4 years, of 13 [5, 21] in 5–24 yo, and of 20 [6, 34] in adults aged ≥ 25 years. Sensitivity analyses demonstrated the robustness of those estimates to alternative assumptions about the number of imported carriers, the duration of carriage, the emergence time of MenW:cc11/SA carriage, and the carriage prevalence in 20–24 yo (Additional file 1: Table S1). Model simulations correctly reproduced the increase of MenW:cc11/SA cases (Fig. 3) and predicted a parallel, albeit much lower, increase of MenW carriage in every age group during the study period (Additional file 1: Fig. S6).

**Table 2:** Parameter estimates (95% CI) in England and in France

Quantity	Symbol	Value in England	Value in France*		
Transmissibility of MenW:cc11/SA (relative to that of non-MenW:cc11)	*r*^(*β*)^	1.20 (1.15, 1.26)	1.15 (fixed)	1.20 (fixed)	1.26 (fixed)
Invasiveness of MenW:cc11/SA in 0–4 yo (relative to that of non-MenW:cc11 in 0–4 yo)	*r*^(*θ*)^_*I*_	4.0 (1.6, 9.7)	7.9 (fixed)	4.0 (fixed)	2.1 (fixed)
Invasiveness of MenW:cc11/SA in 5–24 yo (relative to that of MenW:cc11/SA in 0–4 yo)	*r*^(*θ*)^_*II*_	3.3 (2.1, 5.8)	3.6 (fixed)	3.2 (fixed)	3.3 (fixed)
Invasiveness of MenW:cc11/SA in 25+ yo (relative to that of MenW:cc11/SA in 5–24 yo)	*r*^(*θ*)^_*III*_	1.6 (1.1, 2.3)	1.3 (fixed)	1.5 (fixed)	1.4 (fixed)
Emergence time of MenW:cc11/SA carriage	*t*_*0*_	2009.5 (fixed)	2011.8 (2010.8, 2012.5)	2011.5 (2011.4, 2012.5)	2012.3 (2012.0, 2012.5)
Log-likelihood	–	− 85.5	− 80.4	− 90.5	− 101.8

In England, the parameters were estimated using the iterated filtering algorithm, with the following algorithmic parameters: 150 iterations with 2000 particles, geometric cooling, and random walk standard deviation of 0.2 for each parameter. In both England and France, the log-likelihood of the parameters was calculated as the log of the mean likelihood of 5 replicate particle filters, each with 5000 particles. The log-likelihood profiles associated with each parameter estimate are shown in Additional file 1: Figs. S4 and S5. *MenW:cc11/SA parameters were not re-estimated in France, but fixed at 3 different values in the 95% CI of *r*^(*β*)^ in England

### Emergence of MenW:cc11/SA and model predictions in France

**Fig. 4:**
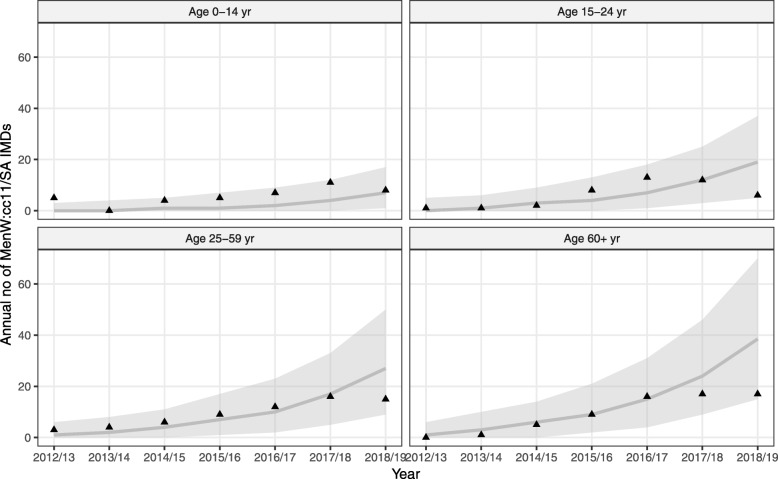
Model fit to MenW:cc11/SA incidence data in France, 2012/2013–2018/2019. The solid lines (envelope) represent the median (95% interval) simulated values from 2000 model simulations; the solid triangles represent the observed data

In France, the first case of MenW:cc11/SA disease was reported in mid-2012 (Fig. 4). However, our estimations suggested that the transmission of MenW:cc11/SA carriage likely started at the end of year 2011 (95% CI [2010.8, 2012.5], Table 2). Model simulations with that estimate correctly reproduced the early increase of MenW:cc11/SA disease in France during 2012/2013–2017/2018 (Fig. 4). However, the model was unable to capture the stagnation or decrease of MenW:cc11/SA cases observed in all age groups in year 2018/2019.

### Simulation study of the duration of silent MenW:cc11/SA carriage transmission

**Fig. 5:**
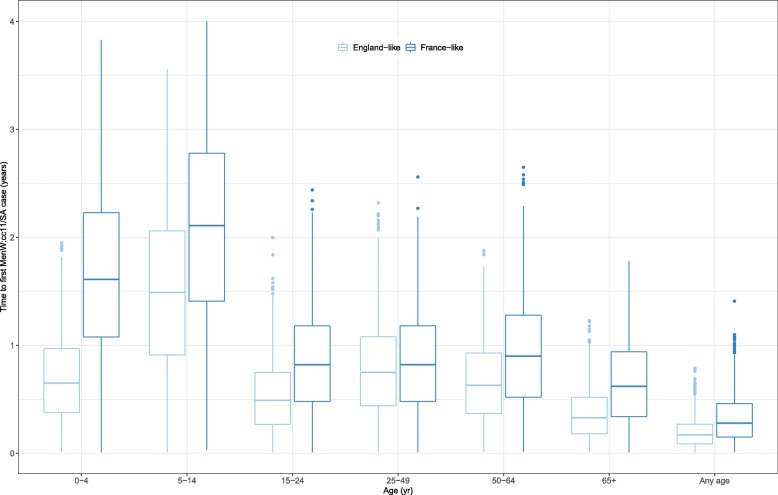
Predicted duration of silent MenW:cc11/SA carriage transmission. Assuming perfect (i.e., complete and not delayed) reporting, we ran 10^3^ model simulations and recorded the time between the start of MenW:cc11/SA carriage transmission and the first report of disease, in a given age group or in any age group. In all simulations, the MenW:cc11/SA parameters used were those estimated in England (Table 2), while the non-MenW:cc11 parameters were fixed to the values calibrated in England (England-like scenarios) or in France (France-like scenarios)

The results presented above suggest that MenW:cc11/SA may spread over long periods of time before the disease is reported. Because of the potential implications for epidemic preparedness, we ran numerical experiments to predict the duration of silent transmission before the first case of MenW:cc11/SA disease, assuming perfect (i.e., complete and not delayed) reporting to the surveillance system. To account for the differences in the epidemiology of MenW between England and France (in particular the lower reported incidence of disease in France, cf. Fig. 2d), we conducted these experiments in both England- and French-like settings. The results, presented in Fig. 5, indicated substantial variability in the predicted duration of silent transmission. In England-like settings, MenW:cc11/SA disease was more likely to be reported first in the age group of 65+ yo (mean [prediction range] time to first MenW:cc11/SA disease case, 0.37 [0.00, 1.33] years) and last in 5–14 yo (1.51 [0.00, 3.56] years). Comparable results were found in French-like settings, although the predicted time to first report was higher (65+ yo, 0.66 [0.00, 1.78] year; 5–14 yo, 2.08 [0.03, 4.00] years). Irrespective of age, the duration of silent transmission was typically a few months, but values exceeding 6 months and up to 17 months were also predicted (English-like settings, 0.19 [0.00, 0.79] year; French-like settings, 0.33 [0.00, 1.41] years).

## Discussion

In this study, we aimed to quantify the transmissibility and the pathogenicity of MenW:cc11/SA, a new strain that has recently caused important shifts in meningococcal epidemiology. To do so, we developed a mathematical model of MenW transmission, carriage, and disease that represented the joint epidemiological dynamics of non-MenW:cc11 and of MenW:cc11/SA strains. Based on detailed incidence data in England, where a marked year-on-year increase of MenW:cc11/SA disease was observed during 2010/2011–2014/2015, we estimated that MenW:cc11/SA strains were 20% more transmissible and 5–20 times more invasive than other MenW strains not belonging to cc11. We also showed that the model parametrized in England correctly reproduced the early increase of MenW:cc11/SA disease in France. Finally, we found that, in both countries, the emergence of MenW:cc11/SA carriage likely started long before the emergence of MenW:cc11/SA disease.

Since its emergence in Latin America, the MenW:cc11/SA strain has spread extensively and has caused increasing endemic disease in many parts of the world [24–26]. Due to its ability to spread, in addition to cause outbreaks and severe disease, it has been hypothesized that the strain was highly transmissible and invasive [29]. Our analysis in England—a country that has conducted rigorous surveillance of meningococcal disease for decades—is consistent with this view and provides precise estimates of MenW:cc11/SA transmissibility and invasiveness. The absence of comparable analyses in other regions, however, limits the comparison with previous studies. Hence, challenge experimental studies in animal models [46] or replicates of our epidemiological analysis in other populations will be useful to assess the validity of our estimates.

The rapidly changing epidemiology of MenW disease in England has been documented in previous studies [24, 27, 28]. After a transient increase in the early 2000s, associated with an outbreak among Hajj pilgrims returning from Saudi Arabia [47], the incidence of MenW disease stabilized at a low annual rate of ≈ 0.05/100,000 [24]. From 2009/2010, however, MenW disease increased steadily and was associated with atypical clinical symptoms and high lethality [48]. Our results confirm that this increase was almost entirely due to the expansion of MenW:cc11/SA, whose incidence almost doubled every year until 2014/2015. The alarming increase in MenW disease prompted the decision to introduce the MenACWY vaccine to immunize adolescents aged 13–18 years in England, with the additional aim of indirectly protecting the wider population through decreased carriage and resultant induction of herd protection [28, 49]. According to national estimates, the vaccine coverage for the school-based program that targeted 13–17 yo was high in every birth cohort, in the range 70–85% during 2015/2016–2017/2018 [50]. In contrast, the vaccine coverage for the general-practitioner–based program that targeted school leavers aged ≥ 18 was lower, reaching only 35–40% by March 2018 [51]. Despite this modest coverage, an early report estimated a 69 [18, 88]% decrease of MenW cases from September 2015 to August 2016 in the first cohort of school leavers targeted by the vaccine [52]. The trends observed in age groups that were not offered MenACWY vaccination also suggested indirect effects of this vaccine [52]. Nevertheless, the effect of the 4CMenB vaccine—which may also confer protection against MenW [53]—offered in infants and toddlers could not be ruled out. Extending the model presented here to incorporate vaccination with MenACWY and with 4CMenB may be useful to refine those vaccine impact estimates and to quantify vaccine effectiveness from the observed incidence data [35].

As in England, the epidemiology of MenW disease has changed substantially since 2000 in France. An increase of MenW disease associated with the Hajj strain was also observed in the early 2000s, but quickly receded and MenW caused few cases during 2006–2011 [23, 31]. Unlike in England, however, new cases of the Hajj strain were reported from 2012, but the increase was transient and mostly associated with recent travel to sub-Saharan countries [54, 55]. Since 2012, the MenW:cc11/SA strain has caused increasing disease and appears to have spread endemically, and sometimes epidemically [23, 56]. Genomic analyses of disease isolates identified both the original UK and the UK 2013 strains, though the latter strain became predominant from 2015 [23]. Our results suggest that, as in England before the vaccines’ roll-out in 2015, MenW:cc11/SA could further spread and cause disease in the years to come. Such trends may undermine the current meningococcal immunization program, which, since January 2018, consists of mandatory vaccination of infants with the conjugate MenC vaccine [57]. Therefore, we propose that mathematical transmission models—such as those developed here or in previous studies [35, 36]—may help forecast the impact of alternative vaccination strategies in France.

An intriguing result of our analyses in France was that the emergence of MenW:cc11/SA carriage may have predated the emergence of MenW:cc11/SA disease by several months. A sensitivity analysis also showed that such a scenario provided an equally good fit to the data in England, although the estimate of MenW:cc11/SA invasiveness was, in this case, lower (Additional file 1: Table S1). Furthermore, our simulation study confirmed the possibility of prolonged and silent transmission of MenW:cc11/SA carriage. These results are consistent with a “tip-of-the-iceberg” phenomenon, which has been described for other infectious diseases that cause infection (or carriage) much more frequently than disease [58]. An important practical consequence is that the absence of MenW:cc11/SA disease does not necessarily imply the absence of MenW:cc11/SA carriage, which may, in fact, already be widespread before the first case report. In a context of international spread and of multi-focal emergence of MenW:cc11/SA, these results may have implications for epidemic preparedness.

Several caveats of our analysis are worth noting. First, we parametrized our model based on the results of a meta-analysis that assessed the overall prevalence of meningococcal carriage [1] and of a clinical trial of meningococcal vaccines in university students in England [43]. Ideally, estimates of MenW carriage prevalence in every age group would be needed to more accurately parametrize our model. Specific data on MenW:cc11/SA carriage would also be needed to verify our model-based predictions of carriage prevalence. Second, it has been proposed that the genetic differences defining the UK 2013 strain—which emerged in 2013 in England—might make it more transmissible and more invasive than the original UK strain [24]. Because of the limited amount of data in England, however, we were not able to examine such differences. Nevertheless, the fact our model’s fit to data did not appreciably worsen after 2013 suggests that such differences may have been modest in England. However, analyzing the data in other countries—such as the Netherlands [24]—where the increase of MenW disease was predominantly caused by the UK 2013 strain may shed more light on this question. Third, the lesser model fit to the most recent MenW:cc11/SA disease data in France suggests that the predictive accuracy of our model might be limited to 5–6 years. Considering the simplicity of our model, however, this forecast horizon is sizable and may still allow to predict the medium-term impact of control interventions (e.g., vaccination) in countries where MenW:cc11/SA started to emerge. Finally, we acknowledge the potential presence of unmeasured confounding factors that may also have been associated with the increase of MenW:cc11/SA in England and in France.

## Conclusions

In conclusion, our study provides the first estimates of the transmissibility and of the pathogenicity of MenW:cc11/SA. Such estimates may be useful to anticipate changes in the epidemiology of MenW and to adapt vaccination strategies. Our results also point to silent, prolonged transmission of MenW:cc11/SA carriage, with potentially important implications for epidemic preparedness.

## Supplementary information

**Additional file 1.** Supplementary Material, composed of Supplementary data, Supplementary methods, Supplementary results, **Figures S1–S6**, and **Table S1.**

**Additional file 2.** Age-stratified annual case counts of MenW:cc11/SA and of non-MenW:cc11 in England and in France.

## Data Availability

The age-stratified annual case counts of MenW:cc11/SA and of non-MenW:cc11 in England and in France are provided in Additional file 2.

## Abbreviations

CC: Clonal complex
CI: Confidence interval
IMD: Invasive meningococcal disease
MenW: Meningococcal serogroup W
MenW:cc11: Meningococcal serogroup W strain belonging to cc11
MenW:cc11/SA: MenW:cc11 South American strain
