# Supplementary material for: Transmissibility and pathogenicity of the emerging meningococcal serogroup W sequence type-11 complex South American strain: a mathematical modeling study
Source: BMC Med. 2020 Apr 22;18:109. doi: 10.1186/s12916-020-01552-7 (PMC7175556; doi:10.1186/s12916-020-01552-7)
Supplement: Supplementary file 1 — Additional file 1. Supplementary Material, composed of Supplementary data, Supplementary methods, Supplementary results, Figures S1–S6, and Table S1. [file 12916_2020_1552_MOESM1_ESM.pdf]

Transmissibility and pathogenicity of the emerging meningococcal serogroup W sequence type-11 complex South American strain: A mathematical modeling study—Supplementary Material

Matthieu Domenech de Cellès, Helen Campbell, Ray Borrow, Muhamed-Kheir Taha, Lulla Opatowski

Supplementary data

Inter-individual contact data Data on age-specific contact rates were available from the POLYMOD study in Great Britain [37]. Because few infants aged <1 yr participated in the POLYMOD study, we used data from a more specific study to fix the pattern of contacts in that demographic [38]. The contact matrix fixed in the model, denoted by $m = (m_{aa'})$, is displayed in Fig. S1.

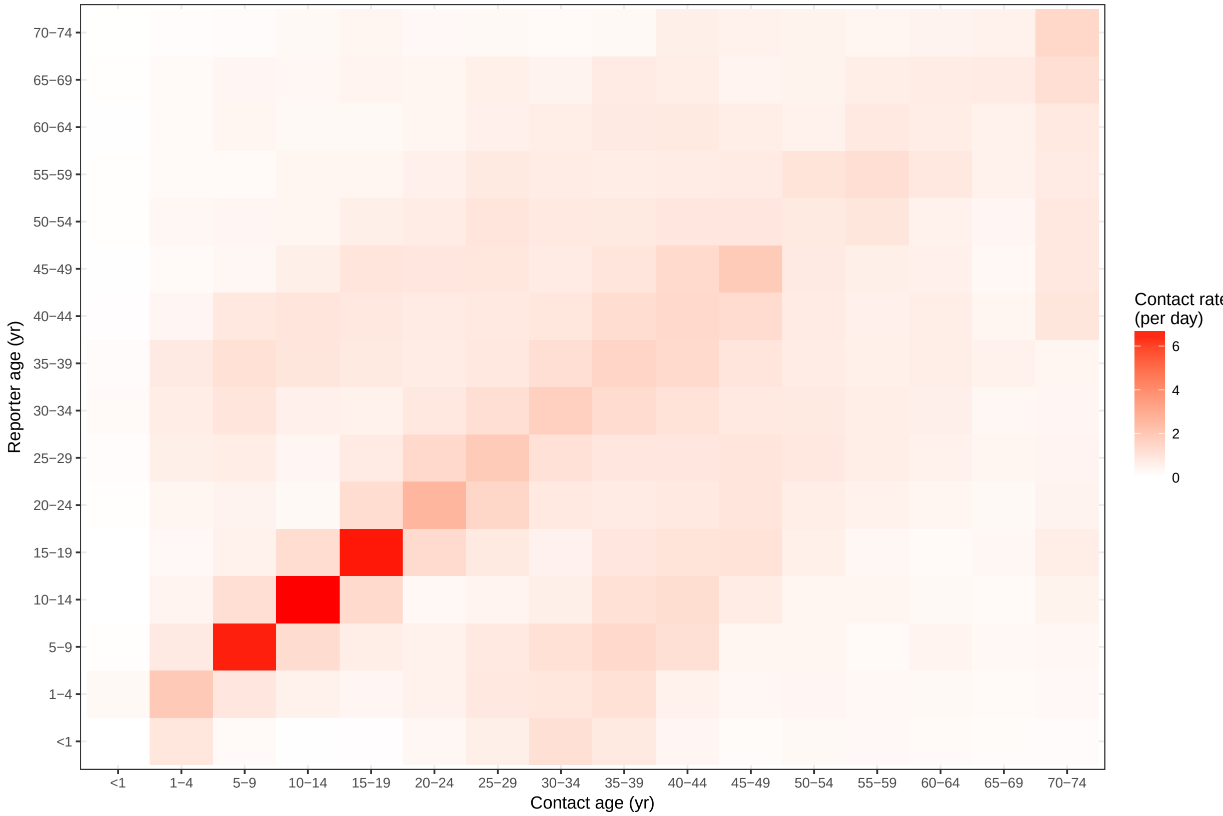

Figure S1: Matrix of age-specific contact rates in Great Britain.

Demographic data The annual number of births and the mid-year age-specific estimates of population sizes during 2009–2016 were available from the Office for National Statistics in England (URL: `https://tinyurl.com/ybzvaomb`; date of access: 15 June 2018) and from the *Institut national de la statistique et des études économiques* in France (URL: `https://tinyurl.com/yabxkoqg` and `https://tinyurl.com/`

yy7lqkmx; date of access: 18 September 2018). The data, presented in Fig. S2, were interpolated to calculate, at every time point, the annual birth rate, $B(t)$, and the age-specific demographic rates, $\mu_a(t)$, so that the simulated population sizes approximately equalled the observed population sizes in every age group.

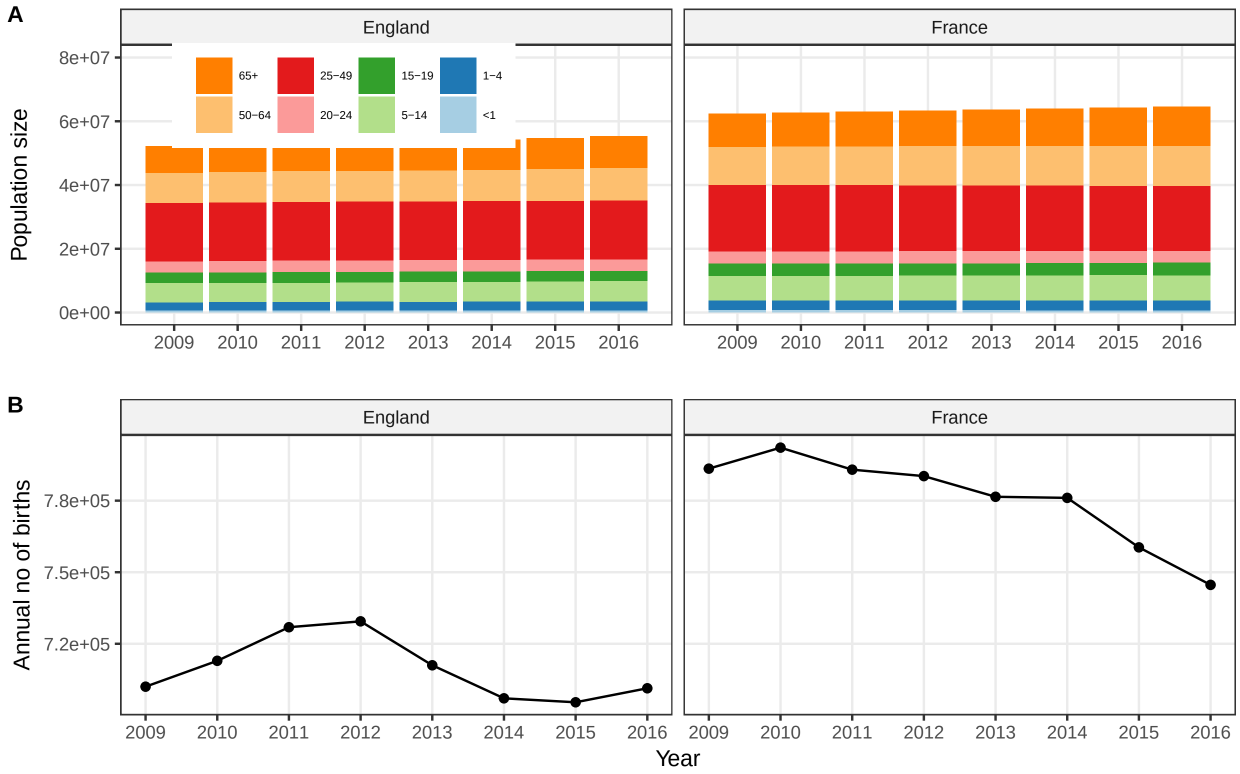

Figure S2: Demographic data in England and in France, 2009–2016. A: Mid-year population estimates. B: Annual no of births.

Supplementary methods

Model formulation

Process model We formulated an age-structured, dynamic model of MenW transmission, carriage, and invasive disease. The model incorporated $A = 16$ age groups: <1, 1–4, 5–9, 10–14, ..., and 70–74 yr. In every age group $a = 0, \dots, A-1$, the population was divided into those individuals not carrying MenW (S_a) and those carrying MenW (C_a). The mean field dynamics were given by the standard Susceptible–Carrier model:

$$\begin{cases} \frac{\mathrm{d}S_0}{\mathrm{d}t} &= B(t) - \lambda_0(t)S_0 + \gamma C_0 - [\delta_0 + \mu_0(t)]S_0 \\ \frac{\mathrm{d}C_0}{\mathrm{d}t} &= \lambda_0(t)S_0 - \gamma C_0 - [\delta_0 + \mu_0(t)]C_0 \end{cases}$$

in infants ($a = 0$) and:

$$\begin{cases} \frac{\mathrm{d}S_a}{\mathrm{d}t} &= \delta_{a-1}S_{a-1} - \lambda_a(t)S_a + \gamma C_a - [\delta_a + \mu_a(t)]S_a \\ \frac{\mathrm{d}C_a}{\mathrm{d}t} &= \delta_{a-1}C_{a-1} + \lambda_a(t)S_a - \gamma C_a - [\delta_a + \mu_a(t)]C_a \end{cases}$$

in older demographics ($a \geq 1$). The parameters δ_a represent the aging rates and γ the carriage clearance rate, such that $1/\gamma$ is the average duration of carriage. The average duration of MenW carriage was fixed to 113 days, the value estimated for serogroups CWY in a controlled trial of meningococcal carriage in university students in England (Appendix Table 3 in [43]). In a sensitivity analysis, we also tested an average duration of carriage of 6 months [35].

Based on previous evidence [24], we assumed that the epidemiology of non-MenW:cc11 did not change during the study period. We then decomposed the total carriage acquisition rate $\lambda_a(t)$ into a component due to the prevalence of non-MenW:cc11 carriers (denoted by $c_a^{(\text{non-c11})}$, assumed fixed) and a component due to the prevalence of MenW:cc11/SA carriers (denoted by $c_a^{(\text{cc11})}(t)$, time-varying):

$$\begin{cases} \lambda_a(t) &= \lambda_a^{(\text{non-cc11})} + \lambda_a^{(\text{cc11})}(t) \\ \lambda_a^{(\text{non-cc11})} &= \beta_a \sum_{a'=0}^{A-1} m_{aa'} c_{a'}^{(\text{non-cc11})} \\ \lambda_a^{(\text{cc11})}(t) &= r^{(\beta)} \beta_a \sum_{a'=0}^{A-1} m_{aa'} c_{a'}^{(\text{cc11})}(t) \end{cases}$$

Here β_a represents the transmissibility of non-MenW:cc11 in age group a and $m_{aa'}$ the rate of contacts between age groups a and a'. To model the emergence of MenW:cc11/SA, we assumed that, from time t_0, a number $\iota = 10$ of imported carriers were present in every age group. This low value was chosen to ensure that the contribution of imported carriers was minimal, in keeping with the observation that most cases of MenW IMDs were not associated with travel or recent entry into the UK during the study period [27]. In sensitivity analyses, we also tested other values of that parameter—spanning three orders of magnitude—to verify the robustness of our results. The prevalence of MenW:cc11/SA carriers was therefore given by:

$$\forall a, c_a^{(\text{cc11})}(t) = \frac{C_a^{(\text{cc11})}(t)}{N_a(t)} = \begin{cases} 0 & , t < t_0 \\ c_a(t) - c_a^{(\text{non-cc11})} + \frac{\iota}{N_a(t)} & , t \geq t_0 \end{cases}$$

where $N_a(t)$ is the time-varying population size in age group a and t_0 is the emergence time of the MenW:cc11 UK strain. Because the first cases of MenW:cc11/SA were reported during the season 2009/10 in England [24], we assumed that MenW:cc11/SA carriage transmission had started at the beginning of that season ($t_0 = 2009.5$). In a sensitivity analysis, we also tested an earlier start time of MenW:cc11/SA carriage transmission

($t_0 = 2008.5$).

Finally, we modeled the dynamics of the cumulative number of IMDs caused by non-MenW:cc11 (denoted by $I_a^{(\text{non-cc11})}$) and by MenW:cc11/SA ($I_a^{(\text{cc11})}(t)$). The equations were:

$$\begin{cases} \frac{\mathrm{d}I_a^{(\text{non-cc11})}}{\mathrm{d}t} & = \theta_a \lambda_a^{(\text{non-cc11})} S_a \\ \frac{\mathrm{d}I_a^{(\text{cc11})}}{\mathrm{d}t} & = r_a^{(\theta)} \theta_a \lambda_a^{(\text{cc11})}(t) S_a \end{cases}$$

where θ_a is the probability of IMD per event of non-MenW:cc11 carriage acquisition, which measures the invasiveness of non-MenW:cc11. The parameter $r_a^{(\theta)}$ represents the invasiveness of MenW:cc11/SA relative to that of non-MenW:cc11 in age group a. A schematic of the model is presented in Fig. S3.

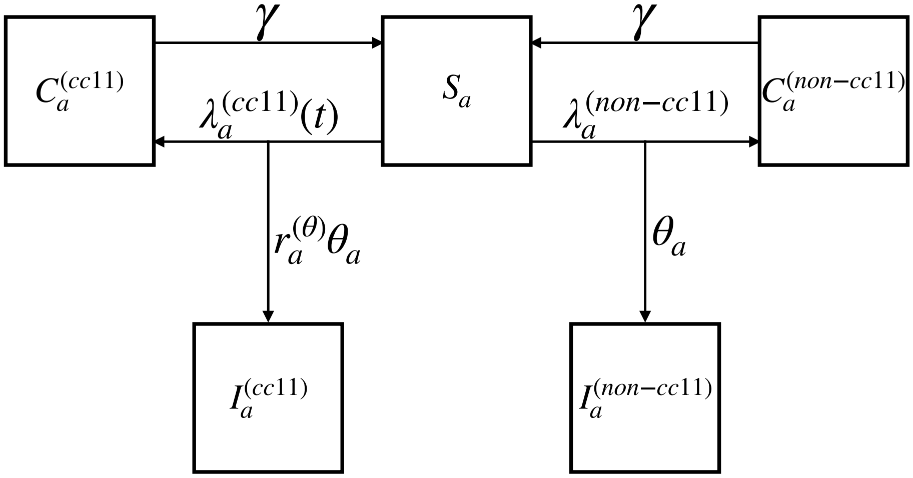

Figure S3: Model schematic. For simplicity, the diagram represents only age group a.

Observation model To complete the model formulation, we modeled the observation process, which relates the model outputs to the observed data and allows to correct for under-reporting. Let

$$I_{a,y} = \int_{y-1}^{y} \frac{\mathrm{d}I_a^{(\text{cc11})}(t)}{\mathrm{d}t} \mathrm{d}t$$

represent the total simulated number of of IMDs caused by MenW:cc11/SA in age group a during the epidemiological year $y = (t-1, t]$. The corresponding annual case report $D_{a,y}$ was modeled as a Poisson distribution:

$$D_{a,y} \sim \text{Poisson}(\rho I_{a,y})$$

where ρ is the reporting probability. We assumed that all cases of MenW IMDs were reported to Public Health England (i.e., $\rho = 1$), based on the results of a previous study that assessed the burden of IMDs by linkage of multiple data sources in England [30].

Model parametrization

Calibration of non-MenW:cc11 parameters

We parametrized the model in two steps. In the first step, we calibrated the transmission (β_a) and the invasiveness (θ_a) parameters so that the simulated dynamics of MenW were stationary before the emergence of MenW:cc11/SA in England. Specifically, we calculated the transmission parameters to reach age-specific targets of non-MenW:cc11 carriage prevalence. Following Argante et al. [35], we defined those targets based on the estimates of the meta-analysis by Christensen et al. [1] (Fig. 2A). Because that meta-analysis assessed the overall prevalence of meningococcal carriage, we then scaled down those estimates so that the carriage prevalence of non-MenW:cc11 was 2% in 20–24 yo (Table 1 in Ref. [43]). The resulting age-specific targets of non-MenW:cc11 carriage prevalence, $c_a^{(\text{non-cc11})}$, are displayed in Fig. 2B. We then back-calculated the values of the transmission parameters β_a, as follows. Let N_a the age-specific population sizes, and μ_a the age-specific demographic rates in 2009 in England. Assuming no demographic changes during the year 2009, the equations for the number of non-MenW:cc11 carriers are:

$$\begin{cases} \frac{\mathrm{d}C_0}{\mathrm{d}t} = & \beta_0 (N_0 - C_0^{(\text{non-cc11})}) \sum_j m_{0j} c_j^{(\text{non-cc11})} - (\gamma + \delta_0 + \mu_0) C_0^{(\text{non-cc11})} \\ \frac{\mathrm{d}C_a}{\mathrm{d}t} = & \delta_{a-1} C_{a-1}^{(\text{non-cc11})} + \beta_a \sum_{a'} m_{aa'} c_{a'}^{(\text{non-cc11})} - (\gamma + \delta_a + \mu_a) C_a^{(\text{non-cc11})} \end{cases}$$

Solving for β_a at equilibrium, we found (Fig. 2C):

$$\begin{cases} \beta_0 = & \frac{(\gamma_0 + \delta_0 + \mu_0) C_0^{(\text{non-cc11})}}{(N_0 - C_0^{(\text{non-cc11})}) \sum_j m_{0j} c_j^{(\text{non-cc11})}} \\ \beta_{a \geq 1} = & \frac{(\gamma_a + \delta_a + \mu_a) C_a^{(\text{non-cc11})} - \delta_{a-1} C_{a-1}^{(\text{non-cc11})}}{(N_a - C_a^{(\text{non-cc11})}) \sum_{a'} m_{aa'} c_{a'}^{(\text{non-cc11})}} \end{cases}$$

To calculate the invasiveness parameters θ_a, we first estimated the average annual incidence (per capita) of non-MenW:cc11 IMDs during the study period, denoted by $\text{Inc}_a^{(\text{non-cc11})}$. Using the values of β_a above, we have:

$$\frac{\mathrm{d}I_a^{(\text{non-cc11})}}{\mathrm{d}t} \approx \frac{N_a \text{Inc}_a^{(\text{non-cc11})}}{\rho} = \theta_a \lambda_a S_a = \begin{cases} \theta_0 (\gamma + \delta_0 + \mu_0) C_0^{(\text{non-cc11})}, & a = 0 \\ \theta_a [(\gamma + \delta_a + \mu_a) C_a^{(\text{non-cc11})} - \delta_{a-1} C_{a-1}^{(\text{non-cc11})}], & a \geq 1 \end{cases}$$

Solving for θ_a, we found (Fig. 2D):

$$\theta_a = \begin{cases} \frac{N_0 \text{Inc}_0^{(\text{non-cc11})}}{\rho(\gamma+\delta_0+\mu_0)C_0^{(\text{non-cc11})}}, & a = 0 \\ \frac{N_i \text{Inc}_i}{\rho[(\gamma+\delta_i+\mu_i)C_a^{(\text{non-cc11})} - \delta_{a-1}C_{a-1}^{(\text{non-cc11})}]}, & a \geq 1 \end{cases}$$

Supplementary results

Log-likelihood profiles The likelihood profiles of the model parameters estimated in England and in France are displayed in Figures S4 and S5. The 95% confidence intervals reported in Table 2 were obtained by smoothing those profiles using local quadratic regression.

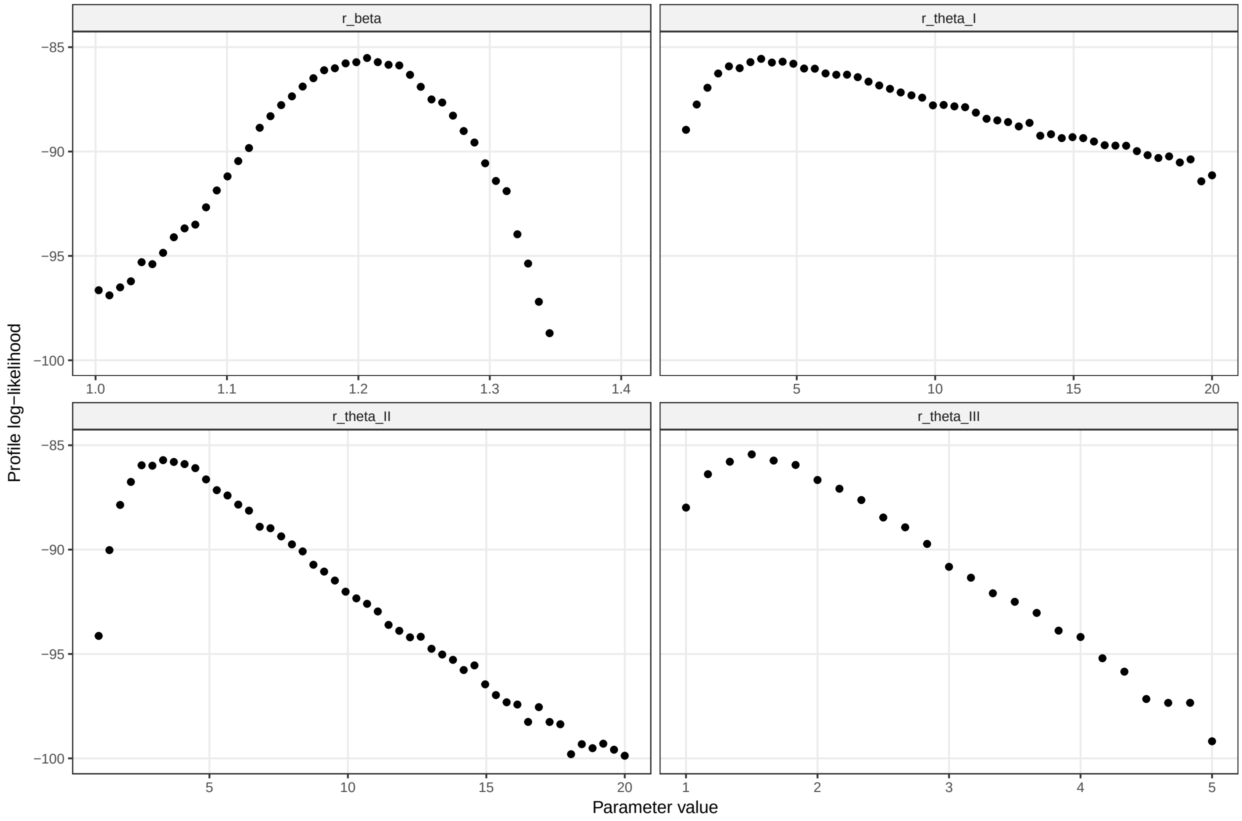

Figure S4: Log-likelihood profiles of parameter estimates in England ($r^{(\beta)}$, $r_I^{(\theta)}$, $r_{II}^{(\theta)}$, $r_{III}^{(\theta)}$).

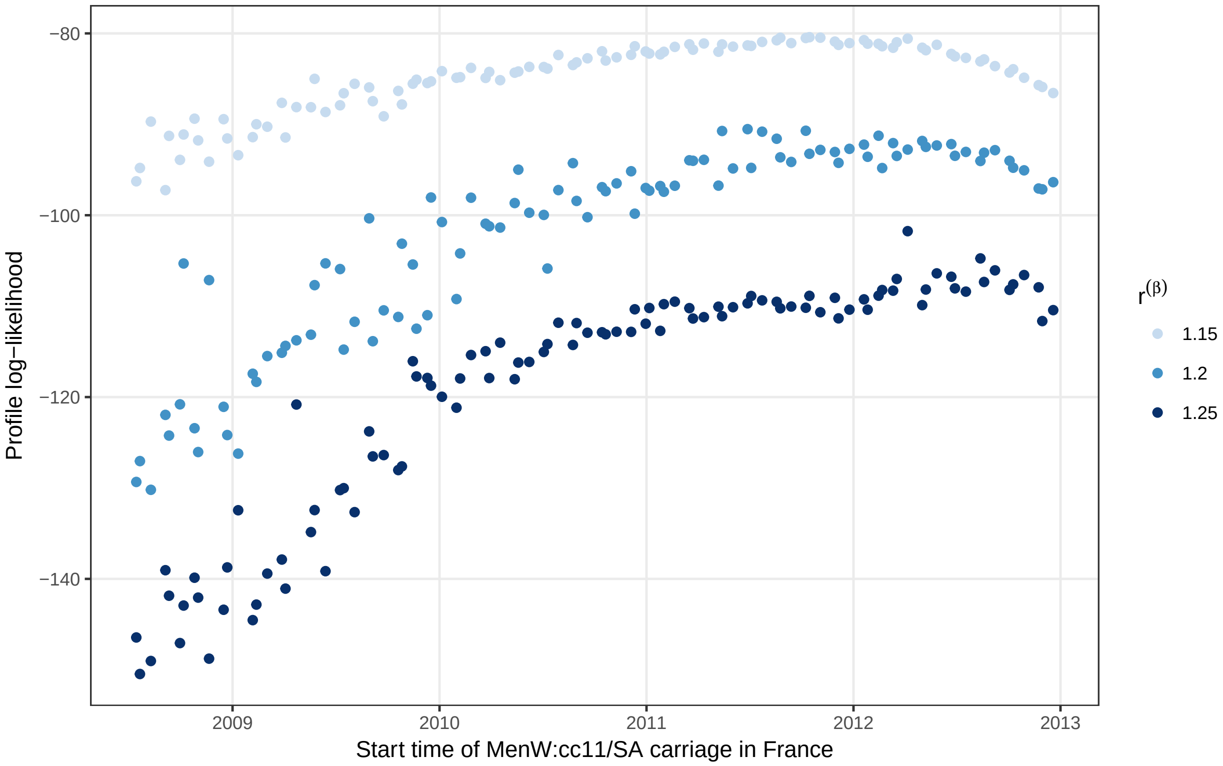

Figure S5: Log-likelihood profile of the start time of MenW:cc11/SA carriage transmission in France. $r^{(\beta)}$: relative transmissibility of MenW:cc11/SA.

Predicted carriage prevalence of MenW

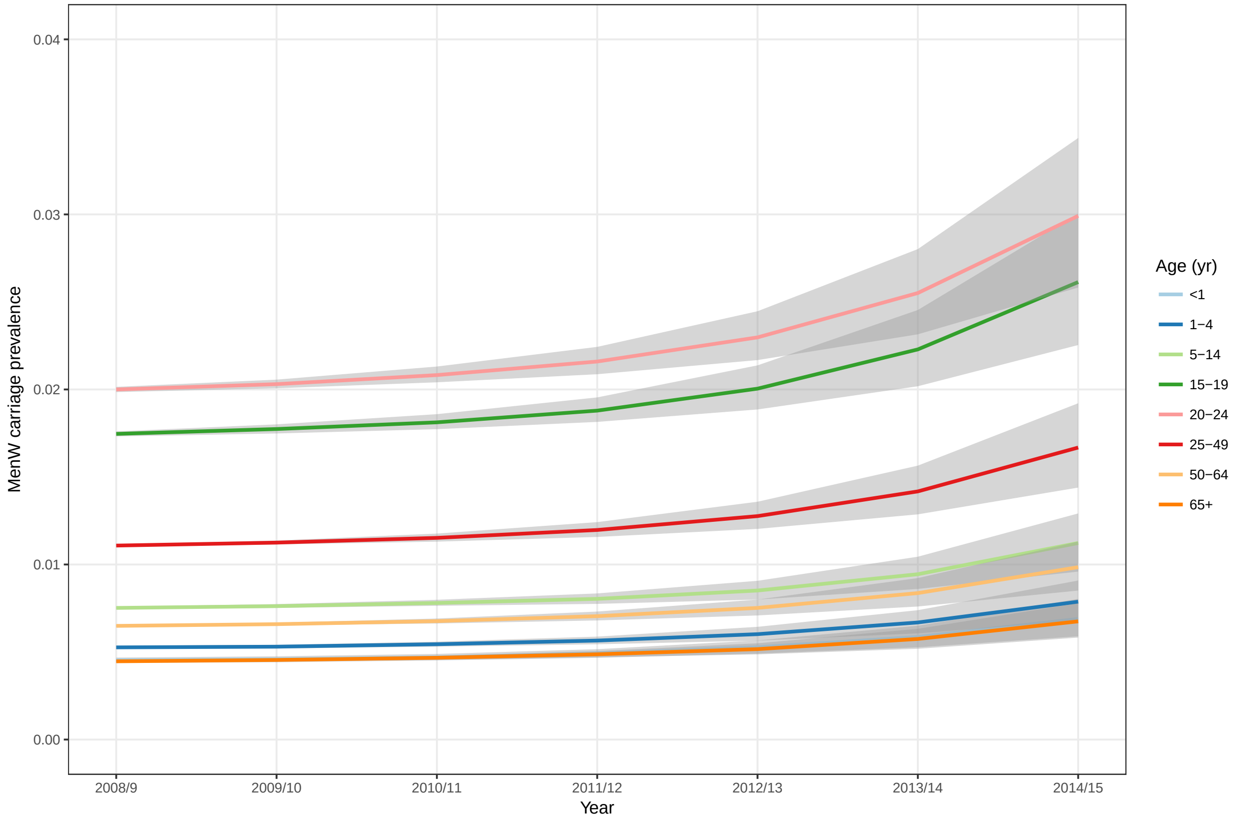

Figure S6: Predicted carriage prevalence of MenW in England. The lines (envelope) represent the median (95% prediction interval) values from 1,000 model simulations.

Sensitivity analyses To assess the robustness of our results, we conducted a number of sensitivity analyses by changing the value of four fixed parameters, namely the average carriage duration ($1/\gamma$), the number of imported carriers (ι), the emergence time of MenW:cc11/SA (t_0), and the carriage prevalence of non-MenW:cc11 in 20–24 yo ($c_5^{(\text{non-cc11})}$). (For the latter sensitivity analysis, we re-calculated the transmission and the invasion parameters of non-MenW:cc11 accordingly.) As shown in Table S1, all the hypotheses tested resulted in comparable parameter estimates and equally good model fit (as judged by the log-likelihood).

Quantity	Base model	Sensitivity analysis 1	Sensitivity analysis 2	Sensitivity analysis 3	Sensitivity analysis 4	Sensitivity analysis 5	Sensitivity analysis 6
Fixed parameters	$\begin{cases} t_0 = & 2009.5 \\ \iota = & 10 \\ \gamma = & \frac{365}{113}\text{yr}^{-1} \end{cases}$	$\gamma = 2$ yr^{-1}	$t_0 = 2008.5$	$\iota = 1$	$\iota = 100$	$c_5^{(non-cc11)} = 0.01$	$c_5^{(non-cc11)} = 0.03$
$\log L$	–85.5 (SE: 0.2)	–85.7 (SE: 0.2)	–85.5 (SE: 0.2)	–85.5 (SE: 0.1)	–85.0 (SE: 0.1)	–85.8 (SE: 0.2)	–85.5 (SE: 0.1)
$r^{(\beta)}$	1.20	1.32	1.21	1.20	1.20	1.20	1.20
$r_I^{(\theta)}$	4.0	6.4	2.1	4.2	2.2	4.5	4.3
$r_{II}^{(\theta)}$	3.3	3.3	3.2	3.3	3.0	3.1	3.2
$r_{III}^{(\theta)}$	1.6	1.5	1.5	1.5	1.7	1.5	1.5

Table S1: Parameter estimates in sensitivity analyses. SE: standard error.
